# The commercialization of biospecimens from Indigenous Peoples: A scoping review of benefit-sharing

**DOI:** 10.3389/fmed.2022.978826

**Published:** 2022-08-04

**Authors:** Tarlynn Tone-Pah-Hote, Nicole Redvers

**Affiliations:** ^1^Department of Family and Community Medicine, University of North Dakota School of Medicine and Health Sciences, Grand Forks, ND, United States; ^2^Department of Indigenous Health, University of North Dakota School of Medicine and Health Sciences, Grand Forks, ND, United States

**Keywords:** Indigenous Peoples, biospecimens, benefit-sharing, commercialization, scoping review

## Abstract

**Background:**

There is ongoing and increasing interest in the commercialization of biospecimen-derived products from Indigenous Peoples. Discourse on benefit-sharing specifically in the context of the commercialization of Indigenous Peoples biospecimens are currently lacking. A better understanding of the potential ethical imperatives is in need of exploration on this emerging topic. This review sought to elucidate through categorization the current discourse in the peer-reviewed literature on the commercialization of Indigenous Peoples' biospecimens from a benefit-sharing perspective.

**Methods:**

A scoping review methodology was utilized to perform a search of PubMed, CINAHL, Embase and Google Scholar. A two-stage screening process was used to assess the relevance of any included articles with subsequent manual open coding of articles. Content analysis was applied to identify the main categories and sub-categories within the article data.

**Results:**

*Thirty-three* articles met the inclusion criteria for analysis. Four overarching categories from the included articles were identified regarding the most common discourse on the commercialization of Indigenous Peoples' biospecimens from a benefit-sharing perspective, including: exploitation through biocolonialism, sovereignty and Indigenous rights, ethical considerations for benefit-sharing, and guidelines and standards concerns.

**Conclusion:**

This scoping review highlighted the crucial need to keep Indigenous communities at the center of research projects, ensuring any benefits, advancement, and potential commercial profits are returned to communities through clear and ethical agreements. We encourage all research institutions and institutional ethical review bodies to better clarify the collective needs and interests of Indigenous communities while centering their sovereignty and rights within the research process as it pertains to potential biospecimen product commercialization.

## Introduction

Indigenous Nations worldwide collectively hold vast knowledges and unique lived experience that roots them directly to the lands they steward. Indigenous Peoples throughout history have been the focus of intrigue for many research traditions, which has led to expressed experiences of harm and exploitation at the hand of researchers and research institutions (1–4). The collective nature of many Indigenous communities since before colonization has led to notions and values around the importance of sharing knowledge, resources, and techniques for the betterment of their communities through collective wellbeing. This collective and sharing nature left many communities vulnerable to the misuse of their knowledge and resources by outsiders.

There have been well-published incidents in the literature whereby Indigenous communities have been at the end of unethical research practices. The Havasupai “diabetes project” collected blood samples for consented research on type II diabetes mellitus, a metabolic condition that devastates many Indigenous Peoples (1). The samples were additionally further used in secondary research such as that on schizophrenia, which participants were not made aware of (1). In 1993, blood samples from a Guaymi Indian woman from Panama were collected who had a gene resistant to leukemia (5). This gene was used for the filing of both United States (US) and international patents on the virus developed from her cell-line (5). This filing led to an uproar from the Guaymi General Congress in Panama, which considered this act to be an invasion of their “genetic privacy” [(5), p. 986]. These two cases are unfortunately only a few of many examples of exploitation of Indigenous communities leading to warranted lack of trustworthiness in research processes and a greater hesitation to participate in research generally (6, 7). Warrented mistrust and hesitation from communities to participate in research is thought to be further amplified when notions or ideas of potential commercialization of biospecimens or genetic materials is possible.

In considering the commercialization process when it comes to products derived from Indigenous communities biospecimens, there is a potential offset between the meanings and ideas of commercial *benefits* for Indigenous communities themselves compared to commercialized entities. Defining what benefits may mean within the Indigenous community context or in the context of partnership (i.e., *benefit-sharing*) is a critical gap in the current dialogues (8). In the benefit-sharing context specifically, it is additionally important to better clarify the positionality of Indigenous communities to eliminate exploitation, while ensuring that benefits are returned to the communities where biospecimens may be sampled from. With this, and in the context of this current work, we define benefit-sharing as:

…the action of giving a portion of advantages/profits derived from the use of human genetic resources to the resource providers in order to achieve justice in exchange with particular emphasis on the clear provision of benefits to those who may lack reasonable access to resulting products and services [(9), p. 207].

It must be noted that in this benefit-sharing definition, the term *genetic resources* is all encompassing, including elements such as traditional plants, foods, and biological material, including, but not limited to, actual genetic material.

Although there has been a developing body of literature exploring research bioethics in the context of Indigenous populations (10, 11), and ongoing discourse around data sovereignty and ownership of biological samples by Indigenous communities (12–14), there has been little direct coverage on the commercialization of products derived from biospecimens from a benefit-sharing perspective specifically. This limited direct coverage of the topic area to date is platformed on an ongoing and increasing interest in biospecimens from Indigenous Peoples for biomedical research and other applications (15–17). Due to this increasing interest in acquiring biospecimens from Indigenous Peoples worldwide for research, medical applications, and potential commercial use, we sought in this scoping review to:

- Elucidate through categorization the current discourse in the peer-reviewed literature on the commercialization of Indigenous Peoples' biospecimens from a benefit-sharing perspective, and
- Reflect on the gaps in the current discourse while considering the need for further policy and research ethics from an Indigenous-centered perspective.

Despite the lack of wider appreciation for Indigenous research methodological approaches, it is additionally important for the authors to position themselves as Indigenous Peoples working with the purpose to improve the health and wellbeing of their communities in this current work. This positionality is becoming an increasing necessity for works that seek to draw conclusions and implications for Indigenous communities (i.e., nothing about Indigenous Peoples, without Indigenous Peoples) (18). With this, the first author is a second-year medical student and an enrolled member of the Kiowa Tribe in the United States and Onondaga from Six Nations, Canada (T.T). The senior author is an enrolled member of the Deninu K'ue First Nation in northern Canada, a clinician, and an Indigenous health scholar (N.R.).

## Materials and methods

The methodology used for this scoping review followed the outline produced by Pham et al. (19) and was based on the framework of Arksey and O'Malley (20) with adjusted recommendations by Levac et al. (21) The scoping review framework was chosen due to the need to keep our scope broad to ensure efficient coverage of the literature for relevant category and gap assessment. The PRISMA-ScR checklist (22) was engaged to ensure appropriate reporting standards for scoping reviews. The study protocol was not registered in the Prospero database, due to Prospero not currently including scoping review protocol registrations.

### Eligibility criteria, procedures, and search terms

With the support and direction of a medical librarian, the search terms and strategy were developed in discussion with the authors (T.T., N.R.). PubMed, CINAHL, and Embase databases were consulted with an initial search date of June 7, 2021, followed by an updated search on November 23, 2021, to identify any additional articles published since the initial search occurred. The search strategy was adjusted as per the needs of the respective database (see Table 1 for an example PubMed search strategy, and *SI Additional file* for the CINAHL, Embase, and Google Scholar search strategy), with no limits on language, the type of publication, or publication dates. Google scholar was searched by consulting the first two pages of the results and then subsequently screening the next two pages until no relevant articles were identified. Manual hand searches of the reference section of key articles were conducted to better ensure all relevant articles were identified in the search. All articles from the search strategy were downloaded and managed within the online database tool specific for review production, Covidence (v2721 a9510157).

**Table 1:** Example PubMed search strategy.

**Database**	**Search Terms**
PubMed MeSH terms	(((“Indigenous Peoples”[Mesh]) OR “Indigenous” OR “first nations” OR “aboriginal” OR “American Indian” OR (“American Native Continental Ancestry Group”[Mesh]) OR “First peoples” OR “Inuit” OR “Maori” OR “Sami” OR “Torres Strait Islanders” OR “American Native Continental Ancestry Group”)) AND ((“biobank*” OR (“Biological Specimen Banks”[Mesh]) OR “stool bank” OR “stored tissue samples” OR “Gene* banks” OR “biospecimen*” OR “biological specimen banks” OR “biological specimen*” OR (“Specimen Handling”[Mesh]) OR (“Medical Waste Disposal”[Mesh]) OR (“Fecal Microbiota Transplantation”[Mesh]))).

Despite our search strategy containing no limits on language, we chose to only include articles in English due to limitations attaining translational support. Only articles published in peer-reviewed journals were included. We additionally only included articles that had coverage and/or discussion on the commercialization of Indigenous Peoples' biospecimens specifically, with no limit on the type of biospecimen relevant in this regard (e.g., blood, stool, etc.).

### Article screening

A two-stage screening process was used to assess the relevance of any included articles according to the previously noted inclusion criteria. A title and abstract screening process was first carried out independently in the Covidence software by two reviewers who were not masked to the article authors or journal name (T.T, N.R). Any discrepancies were resolved by discussion between the two reviewers. Any articles that did not have an abstract were moved on to the next stage of the screening process for further assessment. After the title and abstract review stage, a full text review was completed by two independent reviewers (T.T., N.R.), and any discrepancies resolved by further discussion. Any articles where a full text article could not be attained was removed from the screening process due to the inability to verify the article eligibility criteria.

### Data characterization, summary, and synthesis

All relevant citations were compiled using Mendeley reference manager (version 2.66.0), and subsequent manual open coding of included articles was organized within Microsoft Excel 365 (version 16.56). One reviewer completed the full extraction of the data, and a second reviewer extracted ten percent of the article data to ensure reviewer consistency. Content analysis as specified by Elo and Kyngas (23), and as clarified by Mikkonen and Kaariainen (24) for reviews was completed to identify the main categories and sub-categories within the article data. In cases where there was an overlap between open codes, a majority category was specified after discussion to facilitate organization of the data. A critical appraisal of the included studies was not completed due to the focus of the review on identifying common categories of dialogue within the literature space, independent of any quality parameters of the articles.

## Results

**Figure 1:**
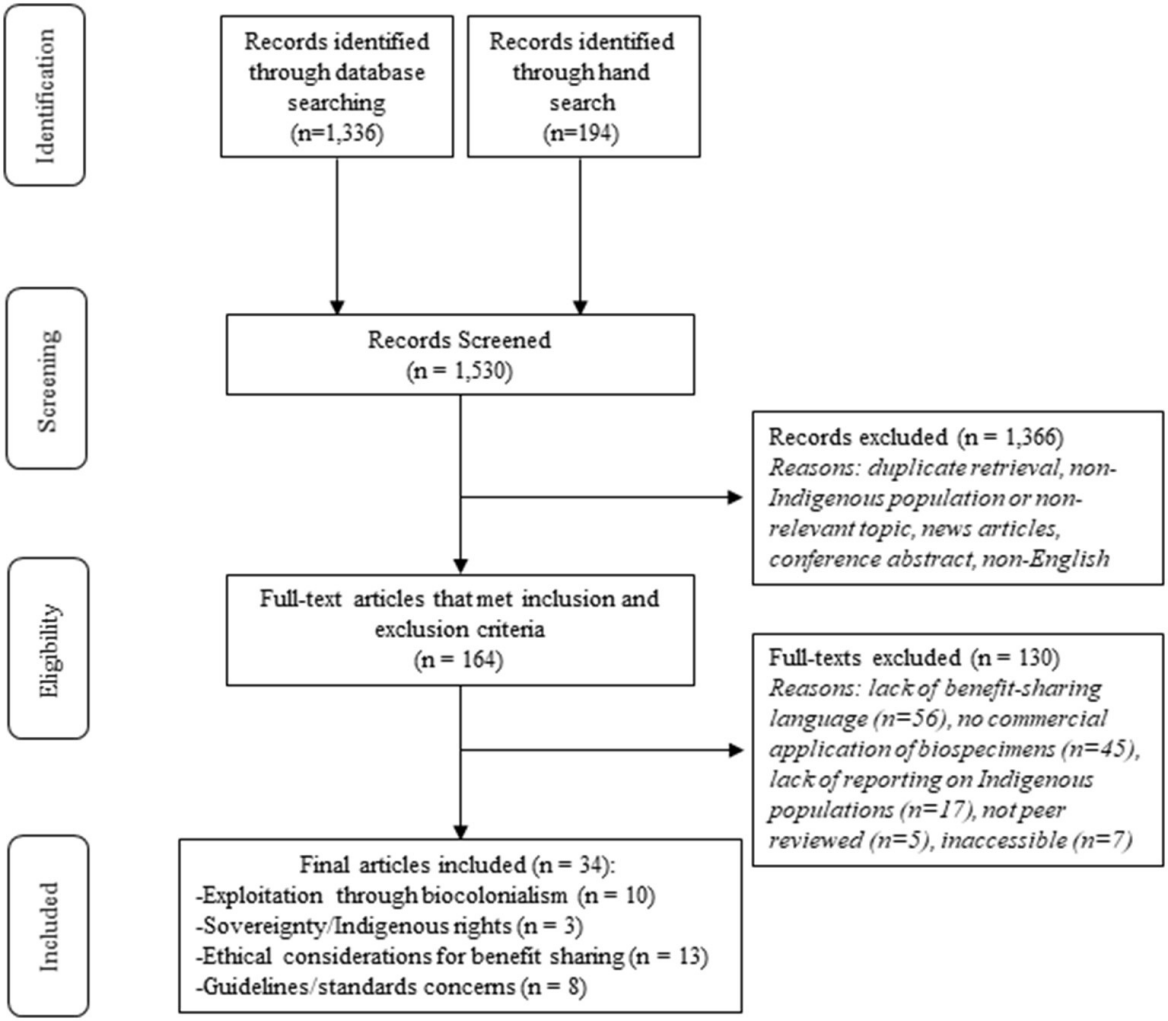
Adapted PRISMA diagram.

A total of 1,530 articles were screened leaving 164 articles for full-text review after duplicates were removed and the title and abstract screen was completed (see Figure 1). After full-text review, 33 articles met the inclusion criteria for analysis (see Supplementary material for full list of included articles).

**Figure 2:**
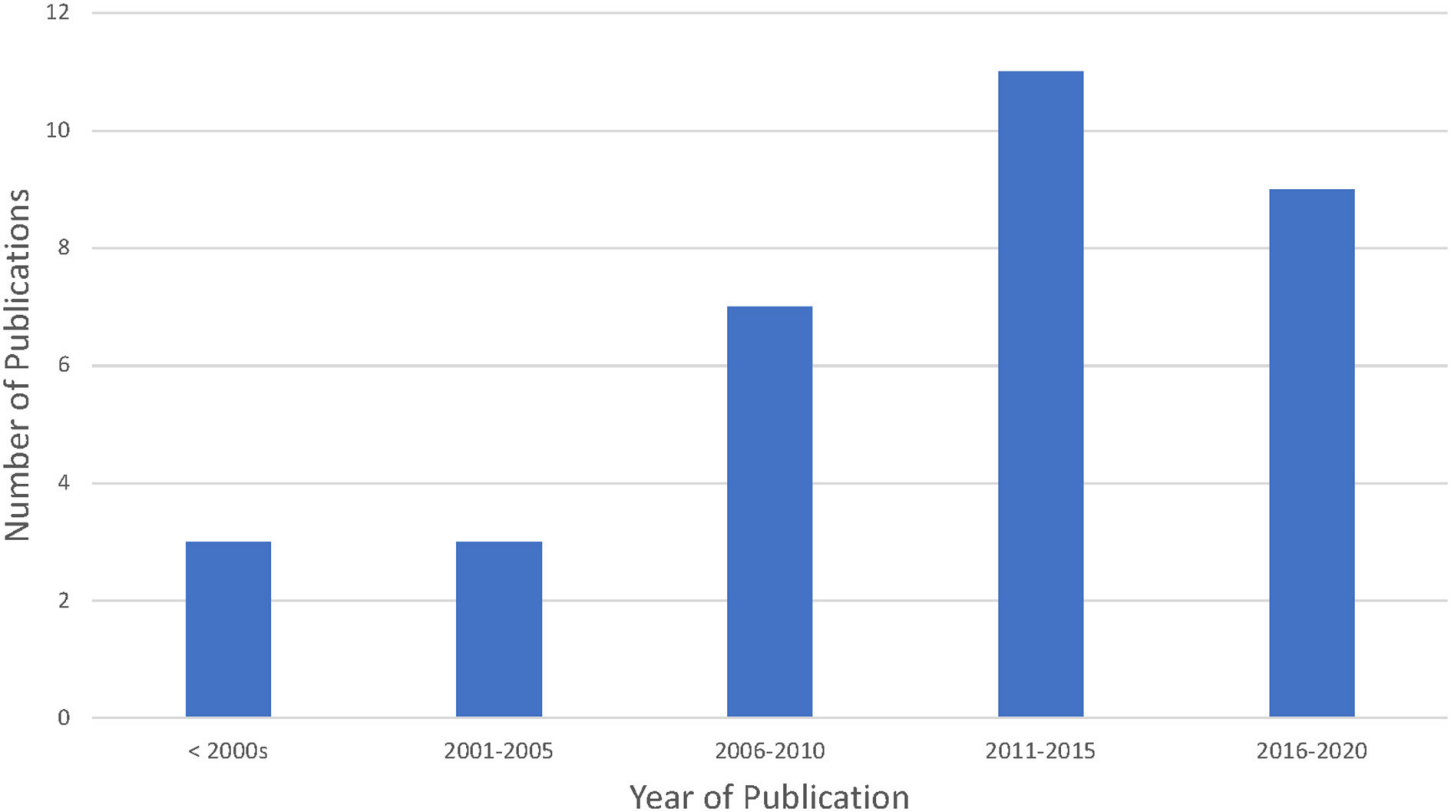
Year of publication for included articles.

The included articles had publication dates ranging between 1998 and 2020 (see Figure 2); however, with the exception of four articles, the majority of papers were published prior to 2017 (average year of publication was 2011, and the median year of publication was 2012). Most articles were a mix of standard journal articles and review papers. The majority of articles were published in three main fields including: law and/or ethics (*n* = 11), policy (*n* = 3) or public health and/or medical journals (*n* = 17). Of the 33 articles included, a majority were published from high income countries (*n* = 24).

Four overarching categories from the included articles were identified regarding the most common discourse on the commercialization of Indigenous Peoples' biospecimens from a benefit-sharing perspective, including: exploitation through biocolonialism, sovereignty and Indigenous rights, ethical considerations for benefit-sharing, and guidelines and standards concerns. Relevant sub-categories were also identified and are discussed within each respective section presented below (see Table 2).

**Table 2:** The main categories and sub-categories identified in the scoping review.

**Categories**	**Sub-categories**
Exploitation through biocolonialism	- Transparency
Sovereignty and Indigenous rights	- N/A
Ethical considerations for benefit-sharing	- Concerns from community
	- Financial, personal, and/or community benefit over corporate benefit alone
Guidelines and standards concerns	- Benefit-sharing plans/contracts
	- Patent considerations

### Exploitation through biocolonialism

*Exploitation through biocolonialism* was a noted category within the included articles (*n* = 10). Biocolonialism can be defined as one variant of neocolonialism in which the relationship of dominance and/or oppression is predicated upon the exploitation of profitable biological material of Indigenous human bodies and/or other living organisms (25, 26). Santos additionally defined biocolonialism to include “commercially-driven…research that secures ownership for profit or academic advancement” [(27), p. 5]. Abayomi et al. defined biopiracy, a concept similar to biocolonialism, specifically as:

…the act of gaining access to biological material…which some academic or commercial benefit may be derived by a technologically advanced country or organization without the intention of fair compensation to the peoples or nations from whose territory the material originated [(28), p. 349].

Throughout this review, many examples were noted where Indigenous Peoples had been subjected to blatant exploitation ranging from the unsolicited use of traditional knowledges for commercial purposes, the extraction of traditional Indigenous medicinal plant knowledge for modern-day pharmaceutical development, or the extraction of varied genetic materials for medical purposes (27–31). It was noted clearly that the exploitation of biospecimens has given rise to hesitation and distrust by Indigenous Peoples to participate in Western medicinal and health research projects (27, 29, 32). With the published reference to Indigenous Peoples as “gold mines for commercial benefit,” [(27), p. 5] without any clear intent of actual defined benefits for and by Indigenous Peoples themselves, a lack of trustworthiness was not surprising (29).

For collective and cultural survival, Indigenous Peoples have shared community resources ranging from food, medicines, and traditional knowledges as previously noted. However, many researchers, seeing these resources as potential avenues for profit, enter communities acting as “vampires” [(32), p. 309] extracting resources with no intent to give back to the community. This has continued to concern Indigenous communities, and have therefore changed the dynamics to the knowledge-sharing process (32, 33). Burhansstipanov et al. highlights how Indigenous Peoples traditional medicines were developed into pharmaceutical agents that sold at a higher price tag can potentially cause the original keepers of those medicines to no longer be able to afford them (30, 34). To some, it has been understood that the use of Indigenous Peoples' resources and knowledge for outside profit and gain at the expense of the community is seen as stealing (35). A few included articles (*n* = 3) noted researchers traveling the world in search of new biological and/or genetic materials they had seen as being potentially profitable (28), and in some cases taking materials without the consent of the Indigenous Peoples from where they originated resulting in exploitation (30, 36).

Issues of *transparency* was a noted sub-category within this exploitation through the biocolonialism category (*n* = 5) (31, 33, 37–39). Many of these articles noted the presence and impact that lack of transparency has had as a part of the exploitation process of Indigenous communities. It was stated that repositories and biobanks that store biological and/or genetic materials need to have clearer statements on the potential commercialization of samples given by donors (31, 33). Harmon et al. (37) mentioned how commercial returns are not always perfectly conveyed to participants or the public and may be an afterthought once samples are taken. Beaton et al. (38) emphasized that the disclosing of potential benefit or commercialization of biospecimens may not be a priority when informing research participants (even when potential profit may be made from samples). A lack of full transparency when it comes to the possibility of commercialization and profit making from donated biospecimen samples is exploitation, potentially further amplified within Indigenous community settings. Foe (39) stated that there may be an assumption made by individuals who voluntarily give samples to biobanks or repositories that their samples will be used for the betterment of society and not for commercial use. Even in cases when individuals may know about the collection of their resources, traditional knowledges, or genetic materials, aspects and prospects for commercialization may not be apparent and can potentially cause research participant hesitation (27, 29, 32).

From the articles reviewed in this category, it was evident that exploitation (as a form of biocolonialism), and a lack of transparency on benefit-sharing and the commercialization of biospecimen samples, was an issue that concerned many Indigenous communities. Impacts on further participation in research endeavors and warranted hesitation to participate in research from a lack of trustworthiness are valid consequences of biocolonialism (31).

### Sovereignty and indigenous rights

*Sovereignty and Indigenous rights*, as it pertains to the commercialization of biospecimens from Indigenous Peoples, was the next category within the included articles (*n* = 3). Although there are no “fixed contours” [(40), p. 73] with regard to the meaning of sovereignty in an Indigenous context, it is generally accepted to mean having:

…the power to create a “safe space” for Indigenous Peoples…ensuring their right of free, prior, informed consent; the right to self-governance; the right to enter into treaties and other agreement; and casting a legal duty on the State to respect, protect and promote Indigenous languages and culture [(40), p. 73].

Sovereignty aims to perpetuate notions of “cultural and legal pluralism”, and is a “source for Indigenous peoples' right to self- determination” [(40), p. 74].

When it comes to the commercialization of and profiting from Indigenous communities' biospecimens, traditional knowledges, or resources, Harry and Kaneche further emphasized the Indigenous Peoples Council on Biocolonialism stance on the inherent rights of an Indigenous Nation “to responsibly enter into any form of commercial or benefit-sharing agreement” [(29), p. 53]. Furthermore, Indigenous Nations may seek to “regulate through taxation, licensing, or other means, the activities of non-members who enter consensual relationships with the Tribe or its members” [(29), p. 39]. Historically, involving the community in any part of the research design outside of the data-collection process was limited. There is evidence of researchers taking control of the research products, including the raw data, living organisms, and genetic information, for commercial and other endeavors that still impact Indigenous communities today (7, 29, 41).

Firm notions of inherent sovereignty govern many Indigenous communities globally, although the right to sovereignty has not been applied evenly by all nation states. Inherent sovereignty is the title that Indigenous Peoples hold when interacting with any other forms of government in addition to institutions (such as research bodies). Due to this, it was emphasized in this review section that there is a critical need for proper protocols and qualified local entities to help oversee the research process given the complex layers of sovereignty and rights inherent within Indigenous spaces (and therefore not leaving everything to researchers and their own ethical bodies) (29, 37). Full Indigenous sovereign control of research processes leaves the opportunity for benefit-sharing and community-driven commercialization within the respective community. Indigenous sovereign control of research processes also ensures clearly defined partnership parameters, rather than parameters developed from a paternalistic relationship where researchers control profitable opportunities at the behest of the communities.

### Ethical considerations for benefit-sharing

Several articles included in this review highlighted discourse around the *ethical considerations for benefit-sharing* (*n* = 12) (8, 30, 31, 39, 42–49). There was a concentration of discussions within two main sub-categories including *concerns from the community*, and, *financial, personal, and/or community benefit over corporate benefits alone*. For the *concerns from the community* sub-category, the concern was centered around the taking of biospecimens for profitability purposes. There was expressed concern from some Indigenous communities that biospecimens, especially those that are unique, could become commodified and commercialized (31, 39, 42). Some communities were hesitant knowing there was prior involvement of commercial companies in biobanking or genetic testing initiatives in general due to the perceived cultural differences regarding commercial applications of research products (31, 43). Others highlighted concerns that private and commercial agendas would overtake and cloud public or community interest, further increasing growing health disparities amongst Indigenous Peoples (31, 44). Many of these concerns appeared to stem from past exploitation and/or unfair commercialization of Indigenous communities biospecimens in the past. There was note additionally, that some of the concern was regarding the lack of return of benefits or profits from biospecimens to the communities which they originated from (31, 45, 46).

For the *financial, personal, and/or community benefit over corporate benefits alone* sub-category, there was a tension between the perceived movement of science towards corporate benefits over and above benefits for communities (either at the financial, personal, and/or community level). One article specifically mentioned that the use of materials for commercial purposes “is seen as unfair because researchers will be profiting from data that was not rightly theirs, nor was given to them” (i.e., advancing the perception of their community materials being “stolen”) [(39), p. 4]. With this, as science has advanced, the idea of pure intellectual curiosity has been said to be clouded, “leaving the public with the impression that scientific information is less a public resource…than a private commodity to fill the coffers of companies and commercial laboratories” [(30), p. 214]. Cunningham (30) further mentioned how commercialization is shaping science leaving a gap in community centered research.

Depending on the institution running the research project (i.e., biobank, university, etc.), the inclusion of commercial involvement and return of benefits could vary substantially of course (47, 48). Ensuring proper protocols, equity, justice, and sharing of profits from samples was stated to begin with continuous communication, informed consent, and direct partnership between researcher and Indigenous communities (8, 46). Ensuring an ethical and co-led system is in place to equitably return benefits to the community while leaving clear options to opt out or reject any sort of commercialization process seemed to be a way to help alleviate concerns on research engagement with communities (8, 49).

### Guidelines and standards concerns

*Guidelines and standards concerns* around benefit-sharing and the commercialization of Indigenous biospecimens were highlighted across several articles included in this review (*n* = 8). There was a clear focus within two notable sub-categories, which included *benefit-sharing plans/contract*s, and *patent considerations*. The need for established Indigenous community-focused research guidelines for working through commercialization and benefit-sharing processes was strongly underscored by several articles; however, additional challenges were presented. As Indigenous Nations often prioritize and uplift their internationally recognized sovereignty (50), the need to have community-defined benefit-sharing and commercialization guidelines and standards in place before research has begun is highly desired by communities (42, 51). In addition, there has been increasing attention on the need for more standardized guidelines to be developed more broadly so there are additional layers of protection for Indigenous Peoples regardless of their local capacity for understanding elements of benefit-sharing and commercialization (46). The need for broader guidelines is seen to be in addition to, and not in isolation of, individual community-led processes that are still important in this context.

Benefit-sharing plans or contracts that clearly outline the research plan are desired to prevent exploitation of those communities participating. This holds true not only in the stages of pre-planning and collection of biospecimens, but also in the post-project plans and dissemination routes, especially if there is potential for commercialization (29, 52). For example, Beaton et al. (8) worked directly with Maori Peoples whose perspective regarding consent, scope, and specificity of the research essentially dictated the use of their samples in all aspects of the research process. For many research projects involving Maori Peoples, contracts identifying how samples may be used by researchers may have to detail the “broad unspecified use, disease specific use, unspecified use, specific project use, use for genetic/genomic analyses, access to clinical records, and/or use for possible commercialization” [(8), p. 347] clearly in the pre-planning phases of the research. Researchers have a stated responsibility to their participating communities' biospecimens (whether for present or future use), to start clear and complete conversations regarding commercialization opportunities early in the research-planning phases (8). Indigenous communities are looking more and more towards regulations for protection, which begins in the form of guidelines and standards.

The potential for restricting the type of research that could be conducted was a noted challenge to benefit-sharing contracts (51, 53); however, other articles referenced some ways in which benefit-sharing plans and guidelines could be implemented successfully within Indigenous communities (54, 55). For example, Ho (54) suggests that agreements between Indigenous communities, research participants, and researchers should follow a set of clear guidelines around benefit-sharing of commercial interests, as well as dissemination of research results. Implementing ethically sound Indigenous-led protocols when working with Indigenous communities with inclusion of benefits beyond only monetary ones such as infrastructure, knowledge sharing and training (56), may better ensure a smoother process throughout the research journey when commercialization is a part of the equation. Another article suggests reconsenting participants when it comes to the commercialization of or secondary research use of biospecimen samples (55). This reconsenting process may be performed individually or on a community-wide level depending on the type of project and commercialization plan (55). Although there have been some suggestions on how to work within Indigenous communities when it comes to creating ethically sound and community-led benefit-sharing plans in the context of biospecimens, there is limited published data available on how such plans can be successfully operationalized.

Lastly, it was evident that the idea of patenting was of concern to some communities and there was question as to whether or not the act of patenting violated the inherent sovereignty held by Indigenous Nations (57). One of the Indigenous global south issues amplified by Wu (5) was regarding the use of patents that could result in commercial products being sold back to low income countries at price tags that they may not be able to afford, while generating great profit to commercial entities. The idea of selling Indigenous Peoples knowledges, medicines, and resources back to them has the potential to cause distress, expand warranted feelings of a lack of trustworthiness, and increasing hesitation for future research participating. Many Indigenous communities see a prime opportunity for exploitation if their biospecimens are patented (7). This opportunity for exploitation has led to some Indigenous groups to create and implement codes and protocol, such as the Tohono O'odham Research code (55), which in part models the Indigenous Peoples Council on Biocolonialism's view on prohibiting the patenting or commercialization of biological materials derived from Indigenous Peoples (29). Despite concerns around patenting, projects such as the Human Genome Diversity Project were not opposed in many cases to the use of patents on human biological material (30). As a result of projects such as this, the impact of patents are still being understood today in the Indigenous context.

## Discussion

Despite ongoing and increasing dialogue within the literature exploring research bioethics in the context of Indigenous populations (10, 11), and ongoing discourse around data sovereignty and ownership of biological samples by Indigenous communities (12–14) (including from Indigenous scholars), there has been little observable investigation regarding benefit-sharing and the commercialization of products derived from biospecimens from Indigenous communities specifically. Our scoping review was an attempt to consolidate the existing discourse to date on this topic to better identify where the literature stands currently and to reflect on any existing gaps.

We situated this work in the global context, anticipating a potential lack of existing literature with explicit coverage of benefit-sharing and/or commercialization on a more local scale. We additionally did not limit the type of biospecimen under consideration within this review (i.e., we included a multitude of potential biospecimens) to ensure a broader potential base of discourse. It became clear throughout our review process that published articles pertaining to benefit-sharing explicitly and/or the commercialization of biospecimens from Indigenous communities was in fact very limited. We were also unclear on how many articles were written by or directly with Indigenous communities, with several articles attempting to generalize to larger groups, regions, or even Indigenous communities globally. Authorship and representation may have affected the narrative of the articles reviewed, and the focus of the content. As previously noted, positionality of authorship is becoming an increasing necessity for works that seek to draw conclusions and implications for Indigenous communities (i.e., nothing about Indigenous Peoples without Indigenous Peoples) (18).

It should additionally be noted that within this review it was clear that potential processes for the commercialization of biospecimens may vary depending on the country and in some cases even the entity involved (e.g., biobank, research institute, etc.) (48). This diversity in potential processes could make it hard to keep track of and ensure equitable benefit-sharing of commercial profits along the research and production chain. However, it was noted clearly and repeatedly that those who should benefit from the commercialization of biospecimens are the people who gave the samples (46, 55). Guidelines are slowly evolving to better ensure communities are included in partnerships and are receiving benefits from their biospecimen samples (49); however, there is much work to be done within this space.

It should also be recognized that throughout this review, it became clear that an absolute definition of biological materials, genetic resources, or biospecimens was in and of itself controversial. Due to the controversy on clear definitions, and a lack of clear boundaries on what constitutes “genetic resources” for example, there is a greater opening and opportunity for potential exploitation. This also makes it difficult to ascertain clear assumptions on what benefit-sharing actually means currently for Indigenous communities.

The Nagoya Protocol is an international agreement that aims to ensure the fair and equitable sharing of benefits from the utilization of genetic resources (also an all-encompassing term in this context) (58); however, many countries, including the US, are not signatories to the Convention on Biological Diversity, which the Nagoya Protocol falls under. This creates legal challenges for ensuring proper protocols are followed in research and commercial settings with regards to benefit-sharing. The Nagoya Protocol has specific mention and protections for Indigenous communities when it comes to their genetic materials (e.g., plants and biospecimens), and their traditional knowledges with the intent to “strengthen the ability of these communities to benefit from the use of their knowledge, innovations and practices” [(58), p. 1]. The Nagoya Protocol may be a good reference to further refine and enhance ethical protections for Indigenous communities in the context of benefit-sharing and commercialization of products derived from biospecimens.

Free, prior, and informed consent (FPIC) is a right and additional mechanism that Indigenous Peoples hold (59); however, institutional ethics review boards rarely consider community-level rights in their deliberations (40). FPIC has not been clearly defined in the research settings, and most often is unknown to ethical review boards in the context of Indigenous Peoples. FPIC is layered on the complexity surrounding how Indigenous sovereignty plays out in the ethics research review process given Indigenous sovereignty's focus on collective rights, as opposed to individual rights and protections inherent within most ethical reviews. In some countries, such as the US, some Indigenous Nations have taken it upon themselves to create additional ethical protections for their community members while ensuring FPIC is upheld at the community level. Tribal institutional review boards are being operationalized to ensure community protections and rights are considered in addition to individual rights and protections (60). These successful Indigenous-led ethics boards, which are themselves rooted directly within Indigenous communities, exemplifies the possibility for collective rights to be considered more broadly in institutional research environments and processes due to many local Indigenous communities having limited oversight capacity.

Other immediate considerations and recommendations for institutional research bodies based on this review, is to support locally led capacity building for Indigenous-run ethics review boards (inclusive of financial and administrative support which can act as barriers for community implementation); ensuring local Indigenous control and/or leadership of research processes; mandating community-directed MOUs with the inclusion of clear line items on potential, current, or future intent or possibility for commercialization; data-ownership agreements; and benefit-sharing agreements where relevant. Indigenous Peoples' past and ongoing negative experiences with Western research practices has created warranted hesitancy to participation, which may in and of itself further contribute to the health disparities experienced by this population. Instituting simple measures as recommended will likely lead to improved environments of trustworthiness, greater self-determination for Indigenous Nations, and increases in the potential for research to directly and meaningfully benefit Indigenous Peoples themselves. Further research is needed to better delineate and clarify concepts of benefit-sharing at the local level within Indigenous communities.

## Limitations

This scoping review was an attempt to elucidate through categorization the current discourse in the peer-reviewed literature on the commercialization of Indigenous Peoples' biospecimens from a benefit-sharing perspective. Given the nature of this review, the number of articles was limited. There is the potential that articles were missed in our search strategy, and therefore this review should not be considered an exhaustive list of all available materials on this topic. Regardless, given the synergy between the articles included for analysis, we feel this review gives a good indication of the common categories of discourse existing on this topic in the existing peer-reviewed literature. We additionally did not perform a critical review of the included full-text articles as a part of this scoping review as our intent was to fully categorize the representative discourse on this topic to date to better inform future research and policy-planning processes.

## Conclusion

This scoping review identified 33 articles with discourse on the commercialization of Indigenous Peoples' biospecimens from a benefit-sharing perspective. There were four overarching categories from the included articles identified: exploitation through biocolonialism, sovereignty and Indigenous rights, ethical considerations for benefit-sharing, and guidelines and standards concerns. This scoping review highlights the crucial need to keep Indigenous communities at the center of research projects, ensuring any benefits, advancement, and potential commercial profits are returned to communities through clear and ethical agreements.

Indigenous Peoples are incredibly diverse, with an extensive heterogenicity in their traditional knowledges and traditional medicines, including an ongoing close connection to the environment. Indigenous Peoples and their lifestyles continue to intrigue many researchers and commercial entities interested in new forms of biological materials. Due to this ongoing and increasing interest, greater protections of Indigenous communities is needed and highly warranted. We encourage all research institutions and institutional ethical review bodies to better clarify the collective needs and interests of Indigenous communities while centering their sovereignty and rights within the research process as it pertains to potential biospecimen commercialization.

## Author contributions

TT-P-H and NR: conceptualization and methodology, data curation, writing—original draft preparation, and review and editing. All authors have read and approved the manuscript.

## Conflict of interest

The authors declare that the research was conducted in the absence of any commercial or financial relationships that could be construed as a potential conflict of interest.

## Publisher's note

All claims expressed in this article are solely those of the authors and do not necessarily represent those of their affiliated organizations, or those of the publisher, the editors and the reviewers. Any product that may be evaluated in this article, or claim that may be made by its manufacturer, is not guaranteed or endorsed by the publisher.
