# Supplementary material for: The commercialization of biospecimens from Indigenous Peoples: A scoping review of benefit-sharing
Source: Front Med (Lausanne). 2022 Aug 4;9:978826. doi: 10.3389/fmed.2022.978826 (PMC9386140; doi:10.3389/fmed.2022.978826)
Supplement: Supplementary file 1 [file Table_1.pdf]

Supplementary Table 1: Master search phrases for PubMed, CINAHL, Embase, and Google Scholar

Database	Search Phrase
PubMed	(((“Indigenous Peoples"[Mesh]) OR "Indigenous" OR "first nations" OR "aboriginal" OR "American Indian" OR ("American Native Continental Ancestry Group"[Mesh]) OR "First peoples" OR "Inuit" OR "Maori" OR "Sami" OR "Torres Strait Islanders" OR "American Native Continental Ancestry Group")) AND (("biobank*" OR ("Biological Specimen Banks"[Mesh]) OR "stool bank" OR "stored tissue samples" OR "Gene* banks" OR "biospecimen*" OR "biological specimen banks" OR "biological specimen*" OR ("Specimen Handling"[Mesh]) OR (“Medical Waste Disposal”[Mesh]) OR (“Fecal Microbiota Transplantation”[Mesh])))
CINAHL	((MH "Indigenous Peoples") OR (MH "Aboriginal Canadians") OR (MH "First Nations of Australia") OR "Indigenous" OR “First Nations” OR (MH "Native Americans") OR (MH "Aboriginal Australians") OR (MH "Alaska Natives") OR (MH "Inuit") OR “Maori” OR “Sami” OR “Torres Strait Islanders”) AND ((MH "Biological Products") OR (MH "Specimen Handling") OR (MH "Tissue Banks") OR (MH "Biological Transport") OR ("Specimen Management") OR (MH "Medical Waste Disposal") OR (MH “Fecal Microbiota Transplantation”) OR "Biological Specimen Bank" OR “stool bank” OR “biospecimen” OR “stored tissue sample” OR “gene bank”)
Embase	('indigenous people'/exp OR ‘ethnic group’/exp OR 'minority group'/exp) AND ('biobank'/exp OR biobank OR ‘biological specimen bank’ OR ‘biospecimen’ OR ‘biological specimen’ OR 'fecal microbiota transplantation' OR ‘stool bank’ OR ‘stored tissue samples’ OR ‘gene bank’ OR ‘genetic procedure’/exp OR ‘waste disposal’ OR ‘biological transport’ OR ‘biological management’)
Google Scholar	("Indigenous" OR "first nations" OR "aboriginal" OR "American Indian") AND ("biobank*" OR "Biological Specimen Banks” OR "stool bank" OR "stored tissue samples" OR "biological management" OR "fecal microbiota transplantation")
